# A New Design Methodology of Asphalt Mixture Dynamic Modulus Based on Pavement Response

**DOI:** 10.3390/ma18133184

**Published:** 2025-07-05

**Authors:** You Huang, Boxiong Feng, Xin Yang, Minxiang Cheng, Zhaohui Liu

**Affiliations:** 1 Engineering Research Center of Catastrophic Prophylaxis and Treatment of Road & Traffic Safety of Ministry of Education, Changsha University of Science and Technology, Changsha 400114, China; hyzju@csust.edu.cn (Y.H.); liuzhaohui@csust.edu.cn (Z.L.); 2 School of Traffic and Transportation Engineering, Changsha University of Science and Technology, Changsha 400114, China; 13842994810@163.com (B.F.); 13627988316@163.com (M.C.)

**Keywords:** road engineering, asphalt pavement, response surface methodology, dynamic modulus, master curve, finite element, mechanical response

## Abstract

The design of asphalt mixture has, for a long time, been an empirical and proof process, causing the mismatch between material design and pavement structure design. To enhance the rationality of asphalt pavement design, this study seeks a path to bridge the gap between asphalt mixture modulus and structural behavior. Firstly, pavement models with different base rigidities, including cement concrete base, cement-treated granular base, and granular base, were constructed to calculate the pavement responses under different dynamic modulus master curve parameters. The influence of master curve parameters on critical pavement responses was identified by the response surface method (RSM). Furthermore, a Whale Optimization Algorithm–Back Propagation (WOA-BP) artificial-neural-network-based pavement response prediction model was established. Then, a database mapping over 100 thousand pavement responses and dynamic modulus master curve parameters was built for determining the dynamic modulus master curve parameters by optimizing the pavement responses. The results show that the impacts of dynamic modulus master curve parameters on critical pavement responses depend on pavement structures. In general, parameter *δ* has the greatest impact, followed by *α*, while the effects of *β* and *γ* are relatively small. The Artificial Neural Network (ANN) performance prediction model, optimized by the WOA algorithm, has a high accuracy. The methodology for determining the dynamic modulus master curve parameter based on the critical response of pavement was successfully implemented. The findings can bridge the gap between material design and structure design of asphalt pavement and provide a basis for more accurate and reasonable asphalt pavement design.

## 1. Introduction

The purpose of asphalt pavement design is to control the critical pavement responses within a reasonable range through the rational combination of structure and material selection. This is undertaken to ensure the service life of the pavement structure [1]. The mechanical empirical method based on the theory of elastic layered systems is the primary approach for asphalt pavement design both domestically and internationally. Under the assumption of linear elasticity, this method assigns a single modulus value to each layer of the pavement structure, disregarding the influences of temperature and load frequency. However, numerous laboratory and field modulus test results indicate that the modulus of asphalt mixtures varies under different loading frequencies and temperature conditions [2,3,4]. Therefore, the modulus values of asphalt mixtures should take into account the influence of both load and temperature. Compared to static rebound modulus, the use of dynamic modulus better reflects the dynamic mechanical characteristics of asphalt mixtures under the combined effects of traffic load and environmental factors.

Dynamic modulus is an essential input for the design of asphalt pavement structures [5,6]. Currently, mainstream asphalt pavement design methods internationally have adopted dynamic modulus as a design parameter. In China, dynamic modulus has also been introduced as a material parameter for pavement structure response calculations in the 2017 edition of the “Highway Asphalt Pavement Design Code” (JTG D50-2017) [7,8]. The dynamic modulus of asphalt mixtures is typically obtained through dynamic modulus tests [9,10]. According to the time–temperature equivalence principle, shifting the dynamic modulus curves obtained at different temperatures and loading frequencies allows the generation of a smooth curve based on a reference temperature. This curve is known as the master curve of dynamic modulus [11,12,13]. Currently, many scholars use an S-shaped function to fit dynamic modulus data, employing the Sigmoidal/Boltzmann function to establish the master curve equation for the modulus of asphalt mixtures [14,15,16]. The master curve of dynamic modulus for asphalt mixtures characterizes the viscoelastic properties of the material. The backpropagation (BP) neural network is a multilayer feedforward neural network based on the error backpropagation algorithm. It has significant advantages in nonlinear mapping, self-learning, adaptability, fault tolerance, and is particularly suitable for solving problems with complex internal mechanisms [17]. In the field of engineering, BP neural networks are commonly used for applications such as strength prediction, modulus inverse calculation, and solving mechanical response [18,19,20]. The introduction of the Whale Optimization Algorithm (WOA) marked a significant advancement in the field of intelligent population-based optimization algorithms. Characterized by its straightforward structure and global optimization capabilities, the WOA has rapidly gained widespread attention in addressing optimization challenges [21]. Notably, it provides a compelling solution to the tendency of BP neural networks to converge prematurely to local minima, a common problem in neural network training. The utility of the algorithm also extends to the field of data computation [22,23,24,25,26,27].

Existing research has mainly been conducted with the help of the dynamic modulus master curve on dynamic modulus influencing factors (temperature, loading frequency, perimeter pressure) and other comprehensive explorations. The main study was carried out on the influence of these influencing factors on the mechanical characteristics laws of the mixture. The study was concerned with the change in asphalt mixture material properties and was not combined with an analysis of the structural mechanical behavior of the pavement. In terms of the characteristic parameters of the main curve, there is no research on the influence of the change in the characteristic parameters of the main curve on the structural mechanical response. At the same time, domestic and foreign research on the design of asphalt pavement structure is also mostly used in a linear elastic laminar theory system, which cannot well describe the mechanical response of asphalt pavement in actual service. China’s current design specification is to select a fixed temperature at a fixed frequency (20 °C, 10 Hz) under the modulus value; however, in service, the asphalt pavement structural layers of the material temperature and stress level will change, which means that as the elasticity is based on the pavement structural analysis of the elastic laminate theory system, selecting a single condition of the modulus value is obviously unreasonable.

**Figure 1:**
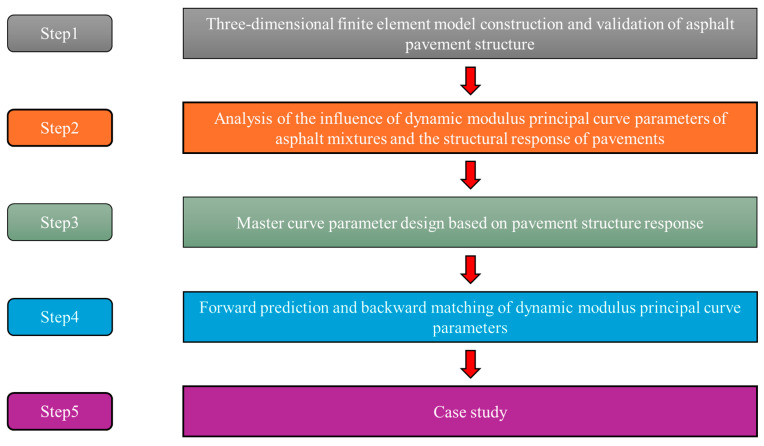
Research idea.

To enhance the rationality of asphalt pavement design, this study seeks a path to bridge the gap between asphalt mixture modulus and structural behavior. Firstly, pavement models with different base rigidity, including cement concrete base, cement-treated granular base, and granular base, were constructed to calculate the pavement responses under different dynamic modulus master curve parameters. The influence of master curve parameters on critical pavement responses was identified by the response surface method (RSM). Furthermore, a Whale Optimization Algorithm–Back Propagation (WOA-BP) artificial-neural-network-based pavement response prediction model was established. Then, a database mapping over 100 thousand pavement responses and dynamic modulus master curve parameters was built for determining the dynamic modulus master curve parameters by optimizing the pavement responses. The findings can bridge the gap between the material design and structure design of asphalt pavement and provide a basis for more accurate and reasonable asphalt pavement design. The research idea is shown in Figure 1.

## 2. Test Methods

### 2.1. Establishment and Validation of Finite Element Models

#### 2.1.1. Finite Element Models

**Figure 2:**
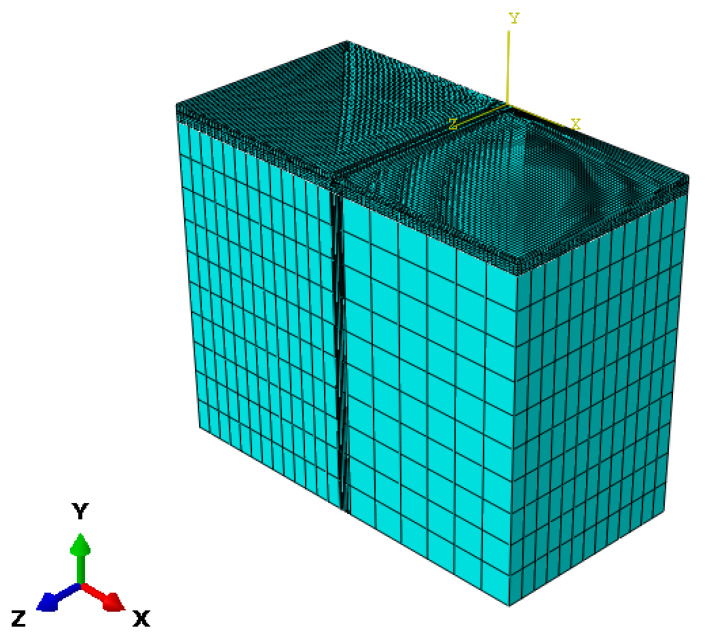
Finite element model of asphalt pavement structure.

Using ABAQUS2022 to establish finite element models of asphalt pavements with different base stiffnesses (rigid (cement concrete base), semi-rigid (cement-treated granular base), and flexible (granular base)). Semi-rigid subbase accounts for 82.6% of highways in China, rigid subbase accounts for 12.3% of heavy-duty roads (ports/mines), and flexible subbase accounts for 5.1% of reconstruction and expansion projects, and these three types of structures cover more than 95% of asphalt pavements in China. Therefore, these three types of structures are chosen as more representative in this paper. The specific configurations of the models are shown in Table 1. In China, there is mostly heavy traffic, so the 8 + 6 + 4 cm structure is mostly used in designing the asphalt layer for highways, i.e., 8 cm for the lower layer, 6 cm for the middle surface layer, and 4 cm for the upper layer. The thickness of asphalt pavement varies for different grades of highways. In this paper, the lower limit value of specification heavy duty road, i.e., 18 cm asphalt layer thickness, is uniformly adopted for the study (JTG D50-2017 Table 5.1.3-1). While a rigid base layer adopting a 30 cm thickness is unified to meet the minimum anti-warping thickness (AASHTO 1993), a semi-rigid base layer and a flexible base layer selecting the 15 cm + 15 cm design are unified to follow the crack-resistant layering construction specification (JTG D50-2017 Article 5.4.3). Following the principle of symmetry, a 3D model of half of the asphalt pavement structure was created with the circular uniformly distributed load diameter as the axis of symmetry. The model dimensions were 9 m × 4.5 m × 20.18 m, and the mesh elements utilized C3D20 (twenty-node hexahedral fully integrated elements). The top surface of the model served as the load-bearing surface, and the DLOAD subroutine was employed to define the load magnitude, application area, and mode of application. Symmetric boundary conditions were applied to the model’s side, constraining normal displacement and symmetric surface rotation. The bottom surface (foundation soil) was subjected to fixed boundary conditions, and interlayer contact was defined as bonded. The mesh division schematic diagram for the Structure 1 model is illustrated in Figure 2 (with certain parts of the foundation structure hidden).

#### 2.1.2. Material Parameters of Pavement Structure

The equation for the master curve of dynamic modulus for asphalt mixtures can be fitted using a Sigmoidal model, expressed as Equation (1):

(1) $$\mathrm{lg}\left|E^{*}\right|=\delta +\frac{\alpha }{1+e^{\beta +\gamma \mathrm{lg}(f_{r})}}$$

where |E*| = the dynamic modulus (MPa); *δ* = the minimum logarithmic value of dynamic modulus; *α* + *δ* = the maximum logarithmic value of dynamic modulus; *β* and *γ* are the coefficients determining the shape of the curve; *f_r_* = the loading frequency at the reference temperature (Hz).

For this paper, a reference temperature of 20 °C and a loading frequency of 10 Hz were chosen. The modulus values of the asphalt surface layer were determined by the master curve of dynamic modulus for the asphalt mixture, with a Poisson’s ratio of 0.35. Other material parameters are listed in Table 2.

#### 2.1.3. FE Model Verification

Taking the three pavement structures shown in Table 1 as examples, with a surface layer modulus of 1400 MPa and other material parameters according to Table 2, a vertical uniformly distributed load (single-axis double-circle, horizontal load of 0.7 MPa, equivalent circle diameter of 0.213 m) was applied to the pavement structure. Structural calculations for the pavement were conducted using both the BISAR3.0 program and the finite element model. The midpoint of the lines connecting the centers of the equivalent circles of the load was selected as a reference point for calculations. The vertical stress and deformation of the asphalt surface layer obtained by the two methods were compared.

### 2.2. Structural Response of Pavements with Dynamic Modulus Principal Curve Parameters

#### 2.2.1. Response Variables for Response Surface Methodology (RSM)

Asphalt mixture dynamic modulus master curve parameters are influenced by various factors such as asphalt material and gradation. Through collecting domestic and international literature [16,28,29,30,31,32,33,34,35], the value ranges for the four parameters of the dynamic modulus master curve of common asphalt mixtures were determined, as shown in Table 3. In this experiment, the number of response variables was *k* = 4, and the axial coordinate value was *α* = 2.

The selection of response values corresponds to the main design indicators of the asphalt pavement structure layer, referring to China Specifications for Highway Design of Asphalt Pavement (JTG D50-2017). The chosen response values were the bottom tensile strain of the asphalt surface layer (*ε*_1_), the bottom tensile stress of the asphalt stabilized layer with inorganic binder (*σ*_1_), and the vertical compressive strain on the top surface of the subgrade (*ε*_2_).

#### 2.2.2. Statistical Experimental Design

According to the CCD model of the response surface methodology, experimental design was conducted using the dynamic modulus master curve parameters (*δ*, *α*, *β*, *γ*) as influencing factors. A total of 30 experimental schemes were designed using the Design-Export software DX12, including 6 groups with repeated experiments at the center points. The specific schemes are detailed in Table 4.

### 2.3. Prediction of Structural Mechanical Responses Based on WOA-BP Neural Network

#### 2.3.1. Database

First, establish the “Dynamic Modulus Main Curve Parameters—Critical Pavement Responses” Database. Establishing finite element models for asphalt pavement structures with three types: rigid base, semi-rigid base, and flexible base. To ensure that the reliability of the model validation carries over to the database predictions and that the results of the parameter sensitivity analyses can be directly applied to the inverse design, the same layer thicknesses as in Table 1 were used for the design. The specific structural configurations are detailed in Table 5. Determine the selection ranges for the parameters *δ*, *α*, *β*, and *γ* of the dynamic modulus master curve by referring to the relevant literature, as shown in Table 6. Adjusting certain parameter values in the experimental scheme outlined in Table 4 by fluctuating within a range of ±5% was employed to expand the dataset. After careful consideration, 145 unique parameter combinations and 10 frequencies were selected, totaling 1450 parameter sets. Computational analysis was conducted on the finite element models of pavement structures corresponding to the 1450 sets of dynamic modulus master curve parameters. The responses of each combination, including bottom tensile strain of the asphalt surface layer, bottom tensile stress of the base layer, and top compressive strain of the subgrade, were extracted. Using the main curve characteristic parameters *δ*, *α*, *β*, *γ*, and frequency f as input variables, and pavement structure response as the output variable, we obtained a “Dynamic Modulus Master Curve Parameters—Critical Pavement Responses” database for three types of pavement structures.

#### 2.3.2. Construction of WOA-BP Prediction Model

The input layer parameters are designated as the four main curve equation parameters (*δ*, *α*, *β*, *γ*) and frequency (*f*). The output layer parameters consist of the maximum tensile strain at the bottom of the asphalt surface layer, maximum tensile stress at the bottom of the base layer, and vertical compressive strain at the top of the subgrade. A three-layer BP neural network is constructed with an input layer consisting of 5 nodes, a hidden layer with 11 nodes, and an output layer with 3 nodes. The training function selected is the Trainlm function, the hidden layer activation function is Tan-Sigmoid, and the output layer activation function is the linear Pureline function. Other neural network parameters are specified in Table 7.

Taking the rigid base layer pavement structure model as an example, data for mechanical responses of pavement structures were collected for 145 parameter combinations across 10 frequencies, resulting in a total of 1450 data points. A random selection of 1160 data points (80%) was chosen as the training set for the neural network, while the remaining 290 data points (20%) were designated as the testing set. Subsequently, the neural network was trained using this dataset.

### 2.4. Design Method and Inverse Matching of Dynamic Modulus Master Curve Parameters

#### 2.4.1. Design Method for Dynamic Modulus Master Curve Parameters

To begin, establish a dynamic modulus master curve parameter database with a sufficient number of samples. The samples should comprehensively reflect various combinations of frequencies and parameters, along with the mechanical responses of pavement structures, to effectively represent diverse pavement materials encountered in real-world engineering projects, ensuring the representativeness of the constructed database. Based on the range of dynamic modulus master curve parameters in Table 6, randomly generate 10,000 sets of parameter combinations. Substitute these parameter combinations under different frequency conditions into the established WOA-BP neural network mechanical response prediction model, yielding predictions for 100,000 response values.

#### 2.4.2. Inverse Matching for Dynamic Modulus Master Curve Parameters

**Figure 3:**
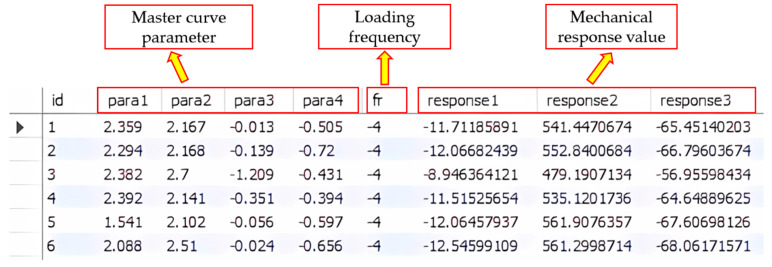
Mechanical Response Data Table.

In this study, MySQL Server 8.0 software was utilized to establish the database, and the prediction data were imported into the database using WorkBench8.0. The data table created for the study is depicted in Figure 3, comprising a total of 100,000 entries. Each entry includes a serial number, main curve parameter combination, loading frequency, and corresponding mechanical response value.

SQL, or Structured Query Language, is a programming language primarily used for database queries and programming. It is mainly applied to data retrieval and the querying, updating, and management of database systems. Utilizing SQL query statements, achieve parameter combination matching based on pavement structure response with the following steps:

(1) Determine the query target: Based on the above analysis, identify the query target as the main curve parameter combination.
(2) Write and execute the query statement: Based on the query target and SQL syntax, write the query statement. Input the query statement into the SQL database management system and execute the query. The query conditions involve mechanical response values, requiring basic query statements, conditional query statements, and grouping query statements.
(3) Analyze the result set: Examine the result set obtained from the query, ensuring it contains the required data and that there are no duplicates or misinterpretations of the data.

### 2.5. Case Study

**Figure 4:**
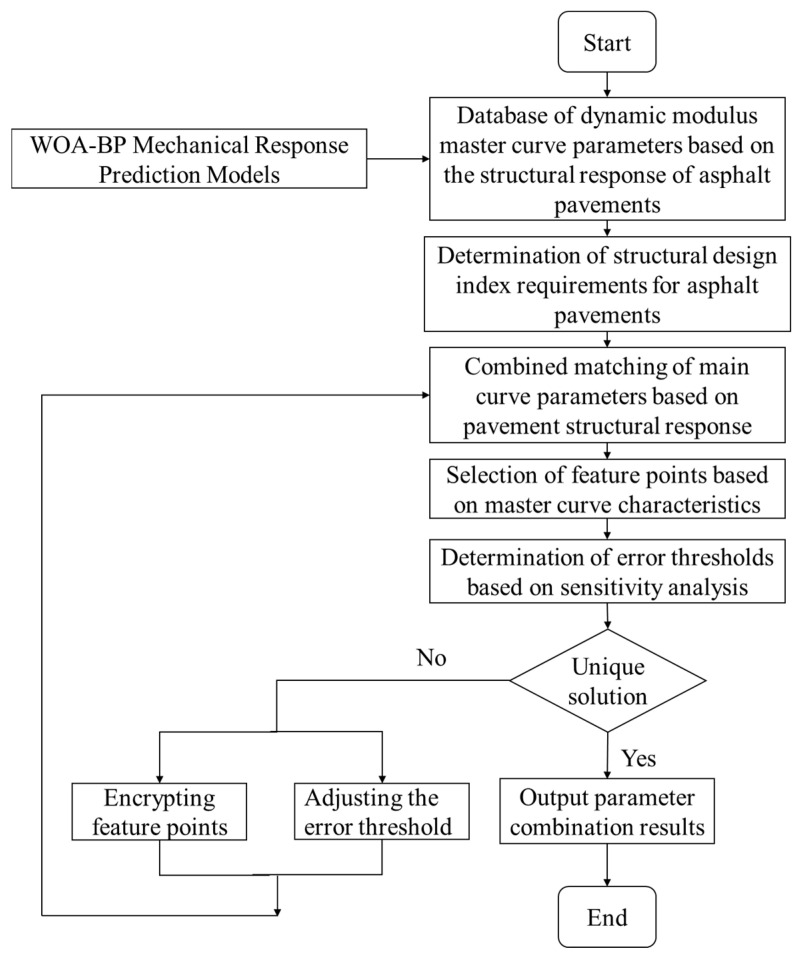
Flow of dynamic master curve parameter design based on pavement response.

To validate the feasibility of the dynamic modulus master curve parameter design method mentioned above, this section intends to solve the dynamic modulus master curve parameters for the asphalt surface layer of three types of asphalt pavement structures: rigid base layer, semi-rigid base layer, and flexible base layer. The analysis process is illustrated in Figure 4, and the specific steps are as follows:

(1) Establish various asphalt pavement structure finite element models, calculate initial data, and use the WOA-BP neural network mechanical response prediction model along with a MySQL database to establish a dynamic modulus master curve parameter database based on the asphalt pavement structure response, comprising a total of 100,000 entries.
(2) Based on the critical pavement responses, strain at the bottom of the asphalt surface layer, tensile stress at the bottom of the base layer, and compressive strain at the top of the subgrade, conduct matching of main curve parameter combinations. Considering the geometric characteristics of the main curve and the results of parameter sensitivity analysis, reasonably select frequency conditions and error thresholds as query criteria. Write SQL query statements to search and match the main curve parameter combinations.
(3) Determine whether step (2) can yield a unique set of dynamic modulus master curve parameters. If the matching result is unique, output the parameter combination result, and the solving process is completed. If the matching result is not unique, optimize the matching strategy using encryption filtering or adjusting error thresholds.

## 3. Results and Discussions

### 3.1. FE Model Verification Results and Analysis

**Figure 5:**
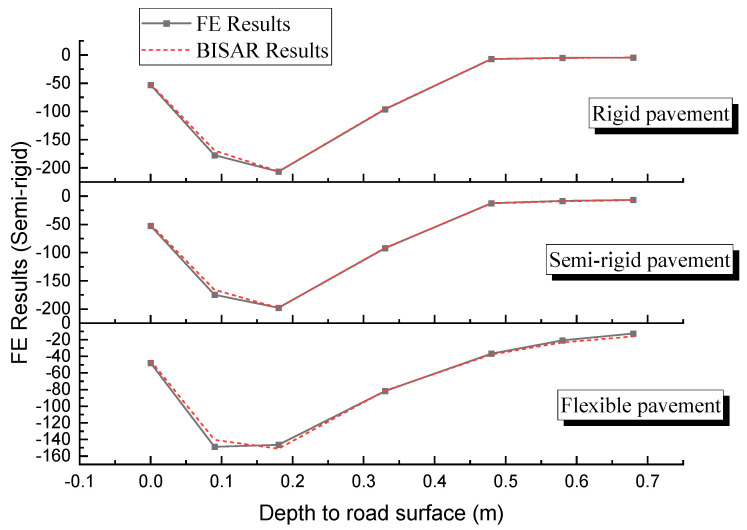
Vertical stresses at different depths.

**Figure 6:**
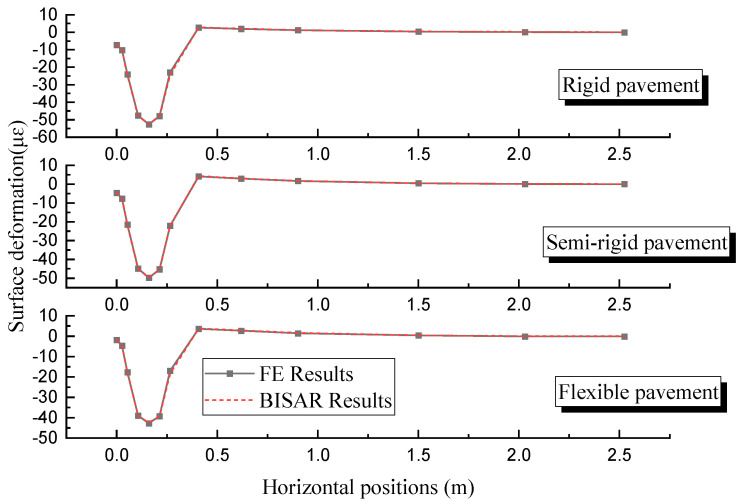
Deformation of the asphalt surface layer.

The finite element calculation results were compared and analyzed with the laminar elasticity theory pavement structure analysis software (BISAR) to verify the reliability of the finite element model. According to AASHTO PP 61-13, the model is judged to be reliable when the error of the strain response (*ε* > 50 με) is less than 10%. In addition, according to NCHRP 1–45, the effect of strain <50 με on fatigue damage is negligible and will not affect the critical response prediction. Also, according to ASTM E2919-22 [36], Standard for Finite Element Modelling of Pavements, the error in stress response should be within 5%. The results are shown in Figure 5 and Figure 6.

From Figure 5 to Figure 6, it can be observed that the finite element solutions for the three pavement structures are generally consistent with the BISAR solutions, with errors mostly within 10%. For some deformation data, the error is greater than 10%, possibly because the calculation point is far from the load, and the deformation of the structural layers is already small. Therefore, the data itself and the error in the calculation process are of similar magnitudes, leading to a relatively large error.

The analysis above indicates that the finite element models of the three pavement structures established in this study are reasonably reliable and can be used for the mechanical response calculations of asphalt pavement structures.

### 3.2. Response Values for RSM

According to the dynamic modulus master curve equation and parameter scheme, the modulus values of the asphalt surface layer under different parameter combinations were calculated and input into the finite element model for pavement structural analysis. The calculation results are shown in Table 8, Table 9 and Table 10. In the tables, the positive and negative signs represent the direction of force: positive indicates tension, and negative indicates compression.

#### 3.2.1. Tensile Strain at the Bottom of the Asphalt Surface Layer

In order to characterize the influence of response variables on the response values of the pavement structure, the data mentioned above were fitted using DX12 software. Subsequently, variance analysis was conducted on the fitting results to explore the extent of the impact of different response variables and their interactions on the response values of the pavement structure.

The asphalt layer bottom tensile strains of three types of pavement structures were fitted by a linear model. The fitting results are shown in Equations (2)–(4). Then, the ANOVA results for the regression models of asphalt layer bottom tensile strain were conducted, and the results are listed in Table 11.

(2) $$\varepsilon_{1R}=-30.38+6.04A+4.46B-2.30C-2.46D$$

(3) $$\varepsilon_{1S}=-50.87+11.81A+8.10B-4.77C-5.21D$$

(4) $$\varepsilon_{1F}=286.49-47.64A-34.10B+20.70C+21.23D$$

where *ε*_1*R*_ = the bottom tensile strain of the asphalt surface layer in the rigid base asphalt pavement; *ε*_1*S*_ = the bottom tensile strain of the asphalt surface layer in the semi-rigid base asphalt pavement; and *ε*_1*F*_ = the bottom tensile strain of the asphalt surface layer in the flexible base asphalt pavement.

**Figure 7:**
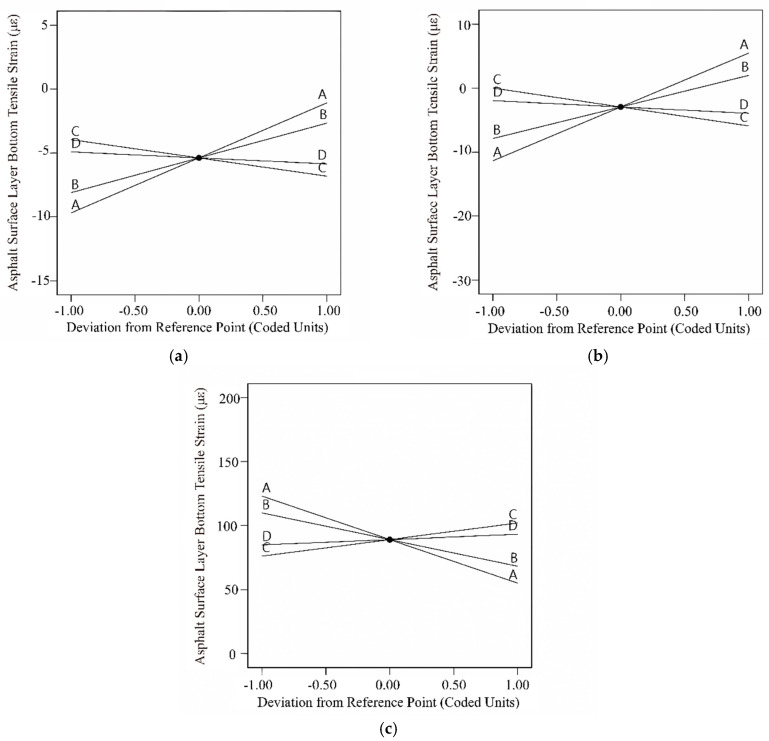
Disturbance curve plot for the asphalt surface layer bottom tensile strain: (**a**) rigid base; (**b**) semi-rigid base; (**c**) flexible base.

The *p*-values are commonly used to assess the significance of a model, usually considering the model significant when *p* < 0.05. From Table 8, it is evident that the *p*-values for the regression models are all less than 0.0001, indicating that the regression models have reached a highly significant level. The correlation coefficients (R^2^) for the three pavement structures are 0.9581, 0.8259, and 0.7912, suggesting a high degree of fit for the regression models, making them suitable for predictive analysis. The F-values for the parameters A, B, C, and D in different pavement structures are ranked as A > B > C > D. This indicates that the impact on the bottom tensile strain of the asphalt layer follows the order *δ* > *α* > *β* > *γ*. In the rigid base pavement, the *p*-values for A, B, and C are all less than 0.0001, signifying a highly significant level; the *p*-value for D is less than 0.05, indicating a significant level. However, in the semi-rigid and flexible base pavements, A and B are highly significant, C is significant, and D is not significant. To visually analyze the impact patterns of different individual variables on the bottom tensile strain of the asphalt surface layer, disturbance curves were plotted. The results are shown in Figure 7.

In Figure 7a, for the rigid base asphalt pavement structure, the bottom of the asphalt surface layer is consistently in a compressed state. The compressive strain at the bottom of the asphalt surface layer decreases with the increase in parameters *δ* and *α* and increases with the increase in parameters *β* and *γ*. In Figure 7b, for the semi-rigid base asphalt pavement structure, when parameters *δ* and *α* are small, the bottom of the asphalt surface layer is in a compressed state, and with the continuous increase in parameters *δ* and *α*, the compressive strain gradually decreases. As parameters *δ* and *α* continue to increase, the bottom of the asphalt surface layer transitions from compression to tension, and the tensile strain continuously increases. In Figure 7c, for the flexible base asphalt pavement structure, the bottom of the surface layer is consistently in a tensile state. The tensile strain values decrease with the increase in parameters *δ* and *α,* and increase with the increase in parameters *β* and *γ*. Additionally, based on the slope of the disturbance curve, it can be observed that parameter *δ* has the greatest impact on the tensile strain value, followed by parameter *α*, and parameter *γ* has the smallest impact. This is consistent with the results of the variance analysis.

#### 3.2.2. Tensile Stress at the Base of the Subgrade Layer

The unbound base layer bottom tensile stresses of three types of pavement structures were fitted by a quadratic multivariate model. The fitting results are shown in Equations (5)–(7). Then, the ANOVA results for the regression models of the unbound base layer bottom tensile stress were conducted, and the results are listed in Table 12.

(5) $$\begin{array}{l} \sigma_{1R}=515.10+70.69A+95.18B-19.76C-22.66D-38.34AB+12.86AC+16.64AD\\ +28.96BC+28.00BD+16.12CD-24.49A^{2}-18.46B^{2}+6.88C^{2}+7.60D^{2} \end{array}$$

(6) $$\begin{array}{l} \sigma_{1S}=643.25-59.08A-35.14B-3.25C+0.37D-8.94AB+4.03AC+6.00AD\\ +30.09BC+27.46BD+16.08CD-6.96A^{2}+1.08B^{2}+12.19C^{2}+14.94D^{2} \end{array}$$

(7) $$\begin{array}{l} \sigma_{1F}=34.52+16.24A+18.49B-4.35C-4.34D-6.31AB+2.21AC+2.60AD\;\\ +3.92BC+3.80BD+2.48CD-3.94A^{2}-3.11B^{2}+0.72C^{2}+0.98D^{2} \end{array}$$

where *σ*_1*R*_ = the bottom tensile stress of the unbound base layer in the rigid base pavement; *σ*_1*S*_ = the bottom tensile stress of the unbound base layer in the semi-rigid base pavement; and *σ*_1*F*_ = the bottom tensile stress of the unbound base layer in the flexible base pavement.

**Figure 8:**
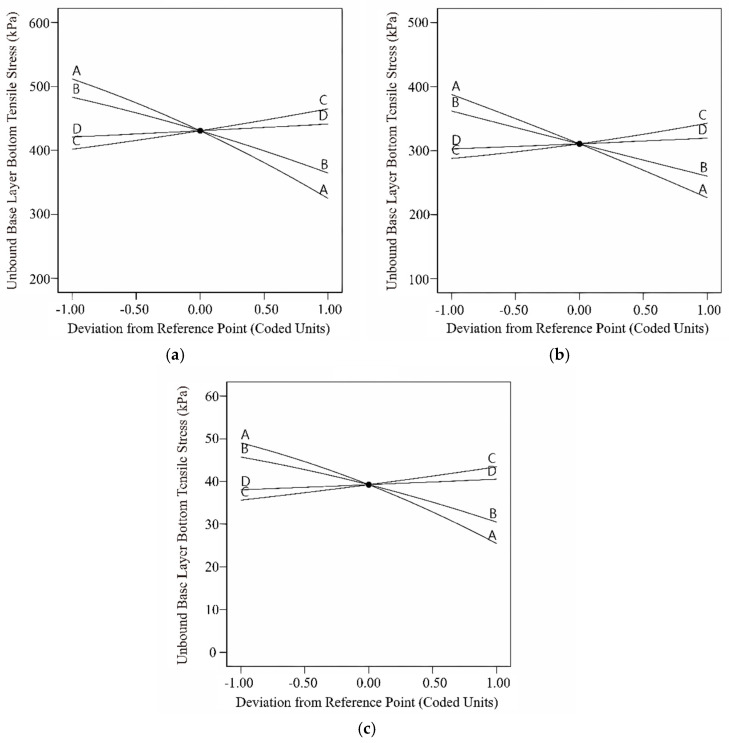
Disturbance curve plot for the unbound base layer bottom tensile stress: (**a**) rigid base; (**b**) semi-rigid base; (**c**) flexible base.

Based on Table 12, the *p*-values for all three models are less than 0.0001, with correlation coefficients of 0.9917, 0.9955, and 0.9952, indicating that the models have reached a highly significant level with a high degree of fit, making them suitable for predictive analysis. In all three pavement structures, the master curve parameters of dynamic modulus significantly influence the bottom tensile stress of the base layer (*p* < 0.05). According to the magnitude of the F-values, the impact order of the four parameters on the bottom tensile stress of the base layer is *δ* > *α* > *β* > *γ*, consistent with the slope magnitudes of the disturbance curves (Figure 8). The bottom tensile stress of the base layer in all three pavement structures increases with the increasing values of parameters *β* and *γ*, while decreasing with the increasing values of parameters *δ* and *α*.

**Figure 9:**
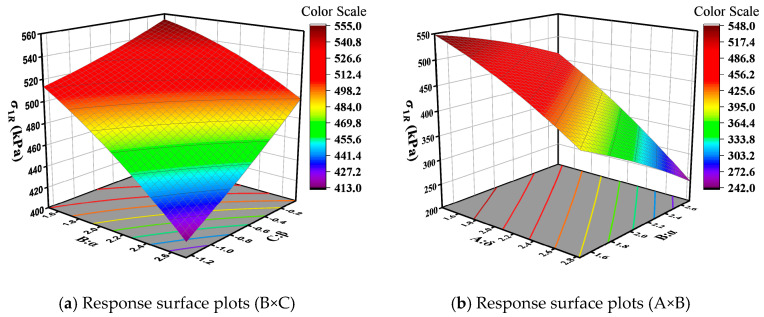
Response surface plots for rigid base.

**Figure 10:**
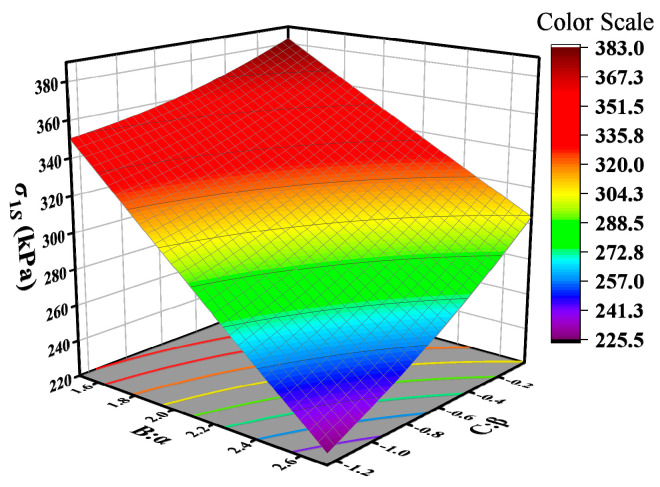
Response surface plots (B × C) for semi-rigid base.

**Figure 11:**
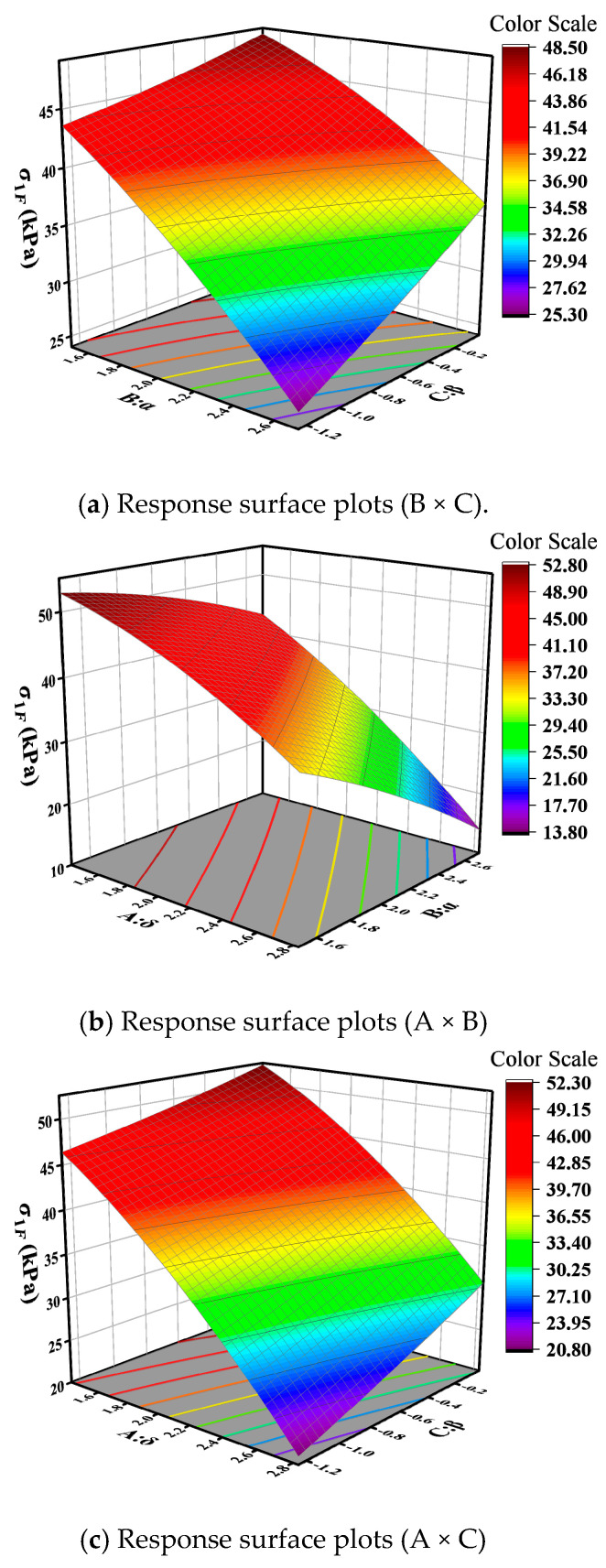
Response surface plots for flexible base.

The impact of interactions between master curve parameters varies with different base layer stiffness: in rigid base layer pavement structures, the interactions of *δ* and *α*, *α* and *β*; in semi-rigid base layer pavement structures, the interaction of *α* and *β*; and in flexible base layer pavement structures, the interactions of *δ* and *α*, *α* and *β*, and *δ* and *β* all exhibit a significant effect (*p* < 0.05). The 3D response surface plots of interaction variables whose *p*-values are lower than 0.05 were drawn, so as to further analyze the effect of the interaction between different variables on base layer bottom tensile stress. The results are displayed in Figure 9, Figure 10 and Figure 11. In the figure, red represents higher response values, while blue represents lower response values. The steeper the slope, the denser the contour lines, and the higher the curvature, the greater the impact of the variables on the response values.

Figure 9a, Figure 10, and Figure 11a illustrate the interactive effects of parameters α and β on the bottom tensile stress in the base layer. The bottom tensile stress decreases along the contour lines in approximately the −31° direction, indicating a decrease in tensile stress as parameters α increase and β decrease. Moreover, the tensile stress is more influenced by parameter α compared to parameter β. The bottom tensile stress in the flexible base layer decreases along contour lines at an angle of 32°, and this trend becomes more pronounced in Figure 9b and Figure 11b. In other words, tensile stress gradually decreases with the increase in parameters δ and α, and the trend accelerates gradually. From Figure 11c, it can be observed that the interaction effect between parameters δ and β is similar to that of parameters α and β. The bottom tensile stress in the flexible base layer decreases along contour lines at an angle of −15°. Additionally, this stress decreases along the direction of increasing δ and decreasing β, and the trend accelerates gradually.

#### 3.2.3. Vertical Compressive Strain at Top Surface of Roadbed

The vertical compression strain on the subgrade surface is also a crucial design parameter for pavement structures. By regulating the vertical compression strain on the subgrade surface, the permanent deformation of the subgrade soil can be controlled. Using a nonlinear multivariate regression model, the fitting results for structural response data of the three types of structures are presented in Equations (8)–(10). Then, the ANOVA results for the regression models of the subgrade surface vertical compression strain were conducted, and the results are listed in Table 13.

(8) $$\varepsilon_{2R}=-122.15+17.98A+13.32B-6.96C-7.32D\;$$

(9) $$\varepsilon_{2S}=-219.98+34.46A+25.11B-13.58C-13.65D\;$$

(10) $$\begin{array}{l} \varepsilon_{2F}=-534.51+83.06A+34.48B-23.29C-24.41D+2.98AB-0.45AC\\ -0.53AD-14.05BC-15.17BD-18.93CD+1.33A^{2}+3.53B^{2}-6.90C^{2}-9.57D^{2} \end{array}$$

where *ε*_2*R*_ = the bottom tensile stress of the unbound base layer in the rigid base layer pavement; *ε*_2S_ = the bottom tensile stress of the unbound base layer in the semi-rigid base layer pavement; and *ε*_2F_ = the bottom tensile stress of the unbound base layer in the flexible base layer pavement.

**Figure 12:**
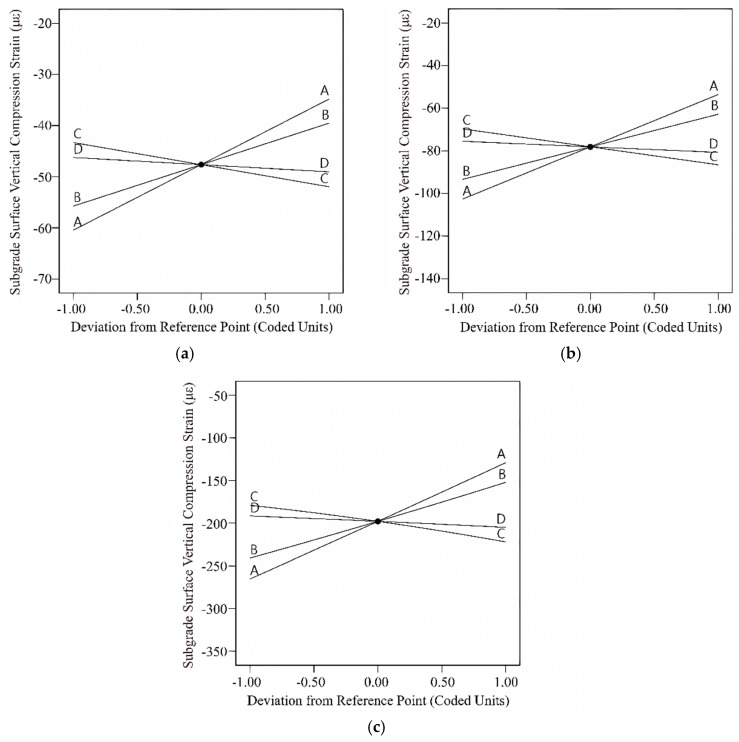
Disturbance curve plot for the subgrade surface vertical compression strain: (**a**) rigid base; (**b**) semi-rigid base; (**c**) flexible base.

From Table 13, it can be observed that the *p*-values for all three models are less than 0.0001, with correlation coefficients of 0.9609, 0.9746, and 0.9998. This indicates that the models have achieved a highly significant level, demonstrating a high degree of fitting and suitability for predictive analysis. From the analysis of the rigid base and semi-rigid base pavement structures, parameters A, B, and C are highly significant, and parameter D is significant. Meanwhile, for the flexible base pavement structure, all four parameters have *p*-values less than 0.0001. According to the F-values, the impact of the four parameters on the vertical compressive strain of the roadbed top surface is ranked as follows: δ > α > β > γ. For rigid base and semi-rigid base pavement structures, the vertical compressive strain of the roadbed top surface is not influenced by the interaction of master curve parameters. However, for the flexible base pavement structures, the *p*-values for BC and CD are less than 0.0001, and the *p*-values for AB and BD are less than 0.05. This indicates that the vertical compressive strain of the roadbed top surface in flexible base pavement is affected by the interaction of various parameter combinations, with the interaction between parameters α and β being the most significant. Figure 12 depicts the single-factor disturbance curve. The impact patterns of the four parameters on the vertical compressive strain of the roadbed top surface are consistent across different pavement structures: compressive strain decreases with an increase in parameters δ and α, while it increases with an increase in parameters β and γ. Analyzing the magnitude of the slope of the disturbance curve reveals that parameter δ has the greatest impact on compressive strain, and parameter γ has the smallest impact.

**Figure 13:**
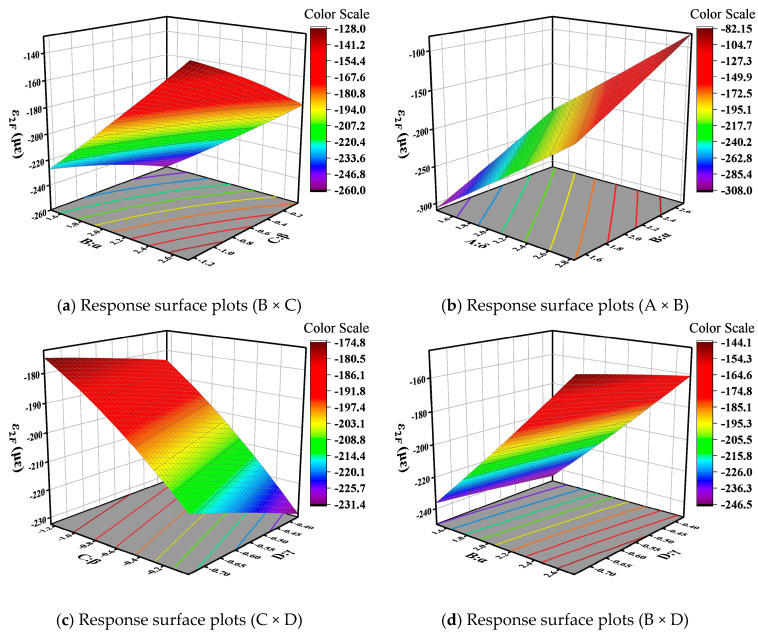
Three-dimensional response surface of flexible base pavement structure.

Based on Equation (10), it can be observed that the vertical compressive strain of the subgrade top surface in the flexible pavement structure is influenced by the interaction of the master curve parameters. Three-dimensional response surface plots were generated for the master curve parameter combinations BC, AB, CD, and BD that significantly impact the pavement structure response, as shown in Figure 13. From Figure 13a, it can be observed that the vertical compressive strain of the subgrade top surface decreases along contour lines at an angle of −23°, indicating a decrease in compressive strain along the direction of increasing parameter α and decreasing parameter β. The interaction effect of parameters δ and α is illustrated in Figure 13b, showing a decrease in compressive strain along the direction of increasing parameters δ and α. Figure 13c depicts the interaction analysis of parameters β and γ, showing an increase in compressive strain along contour lines at an angle of 18°. An increase in parameters β and γ leads to an increase in compressive strain on the top surface of the subgrade. As seen in Figure 13d, the interaction effect of parameters α and γ is similar to that of parameters α and β. Compressive strain decreases along the direction of increasing parameter α and decreasing parameter γ, with the influence of parameter α being higher than that of parameter γ.

#### 3.2.4. Sensitivity Analysis

In order to quantitatively analyze the impact of parameter fluctuations on the mechanical response of asphalt pavement structures, the relative sensitivity coefficient *S* is defined as in Equation (11).

(11) $$S=\left(\frac{\Delta R}{R}\right)/\left(\frac{\Delta P}{P}\right)$$

where *R* represents the model output (pavement structure response); *P* represents the model input (master curve parameters), taken at the central values within the parameter domain; Δ*R* is the change in the model output result; and Δ*P* is the change in the parameters.

When varying the parameters *δ*, *α*, *β*, and *γ* by ±10% around their central values and inputting them into the finite element model, the changes in pavement structure response were computed. The sensitivity coefficients (S) for three critical mechanical responses of the pavement structure are presented in Table 14. The sign (positive or negative) of S in the table indicates the correlation between parameter fluctuations and response changes: a positive sign denotes a positive correlation, while a negative sign denotes a negative correlation. The magnitude of |S| reflects the strength of the parameter sensitivity, with larger values indicating stronger sensitivity. Taking the example of the sensitivity of the bottom tensile strain of the asphalt surface layer to the parameter *δ* in a semi-rigid base pavement structure, the corresponding sensitivity coefficient (S) is 47.19. This indicates that when the parameter *δ* increases by 10%, the bottom tensile strain of the asphalt surface layer increases by 472%.

For both rigid and semi-rigid base asphalt pavements, the sensitivity to variations in main curve parameters is as follows: the bottom tensile strain of the asphalt surface layer exhibits the highest sensitivity, followed by the vertical compressive strain on the subgrade top surface, and the lowest sensitivity is observed in the bottom tensile stress of the base layer. Conversely, for flexible base asphalt pavements, the sensitivity to the four parameters is as follows: the bottom tensile strain of the asphalt surface layer has the highest sensitivity, followed by the bottom tensile stress of the base layer, and the vertical compressive strain on the subgrade top surface exhibits the lowest sensitivity. For the same type of structural response, the absolute magnitude of sensitivity coefficients to variations in main curve parameters follows the order *δ* > *α* > *β* ≈ *γ*.

The sensitivity of structural response to variations in main curve parameters is influenced by the combination of pavement structure. In rigid base asphalt pavements, where the surface layer is in a compressed state and primarily serves to transmit loads, an increase in parameters δ and α, and a decrease in β and γ, result in an increase in the surface layer modulus, enhancing its deformation resistance and reducing compressive strain. Therefore, the bottom tensile strain of the asphalt surface layer in rigid base structures is negatively correlated with parameters δ and α, and positively correlated with parameters β and γ. In flexible base structures, where the surface layer is the primary load-bearing structural layer and is predominantly in tension, an increase in the surface layer modulus enhances its deformation resistance, resulting in a decrease in structural response. Therefore, the bottom tensile strain of the asphalt surface layer in flexible base structures is negatively correlated with parameters δ and α, and positively correlated with parameters β and γ. For semi-rigid base structures, when parameters δ and α are small, the asphalt surface layer is in a compressed state, resulting in an increase in the surface layer modulus, enhanced deformation resistance, and reduced compressive strain. Therefore, parameters δ and α are negatively correlated with the bottom tensile strain of the asphalt surface layer, while parameters β and γ are positively correlated. However, as parameters δ and α continue to increase, the bottom of the asphalt surface layer transitions from compression to tension. In this case, parameters δ and α become positively correlated with the bottom tensile strain of the asphalt surface layer, while parameters β and γ become negatively correlated with tensile strain. For the bottom tensile stress of the base layer and the vertical compressive strain on the subgrade top surface, an increase in the surface layer modulus reduces the stress transferred from the surface layer to the lower layers of the structure, resulting in a corresponding decrease in response. Therefore, both are negatively correlated with parameters δ and α, and positively correlated with parameters β and γ.

### 3.3. Results and Analysis of Structural Mechanical Response Prediction Based on WOA-BP Neural Network 

**Figure 14:**
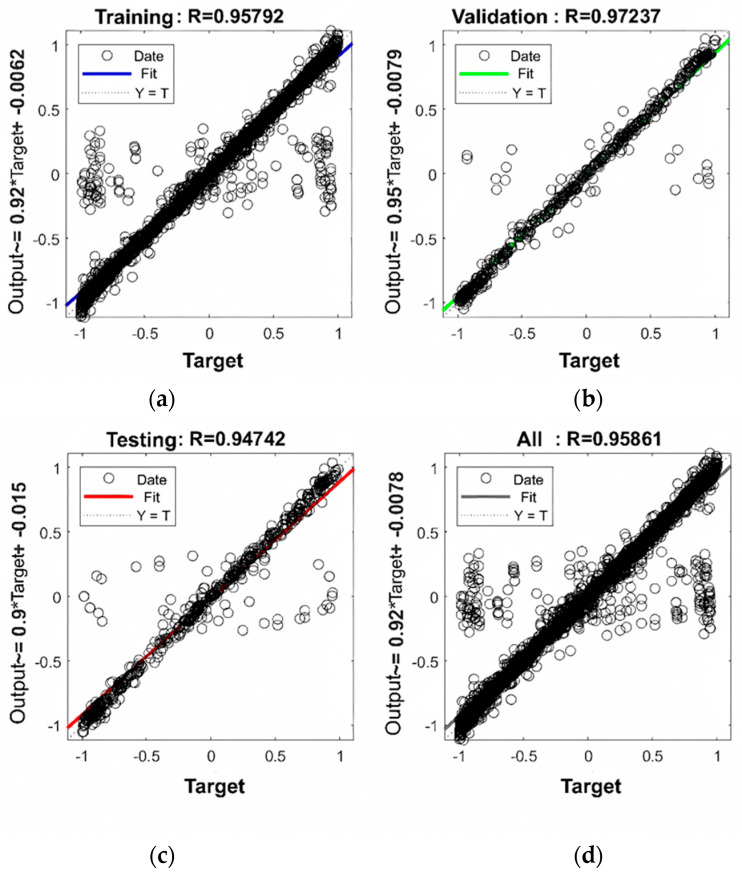
Training results of WOA-BP prediction model for mechanical response of rigid base pavement structure: (**a**) training samples; (**b**) validation samples; (**c**) testing samples; (**d**) overall samples.

**Figure 15:**
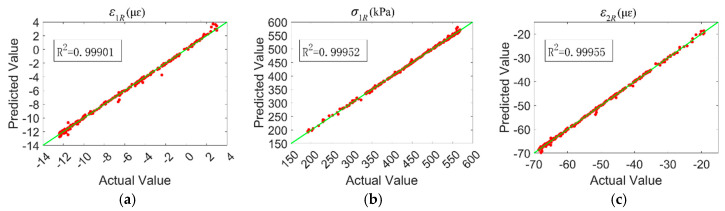
Structural mechanical response of rigid base pavement WOA-BP prediction results: (**a**) asphalt surface layer bottom tensile strain; (**b**) unbound base layer bottom tensile stress; (**c**) subgrade surface vertical compression strain.

The training results of the rigid base layer pavement structure mechanical response prediction model based on the WOA-BP neural network are illustrated in Figure 14. In Figure 14, the correlation coefficients (R) for the training, validation, testing, and overall samples of the WOA-BP neural network are 0.95792, 0.97237, 0.94742, and 0.95861, respectively. All correlation coefficients are greater than 0.9, indicating the suitability of the model for predicting the mechanical response of asphalt pavement structures. Figure 15 shows the prediction results of the WOA-BP predictive model for the mechanical response of the rigid base layer pavement structure on the testing dataset. From the distribution of the data points in the graph, it can be observed that the model’s data points are concentrated, indicating good predictive performance of the model.

The prediction results for the semi-rigid base layer and flexible base layer asphalt pavement are similar, and the model’s predictive performance is presented in Table 15.

From Table 15, it is evident that the sample correlation coefficients of the asphalt pavement structure mechanical response prediction model, optimized by the WOA algorithm, are all greater than 0.9. This indicates that the WOA-BP neural network can more accurately handle multidimensional variables. The asphalt pavement structure mechanical response prediction model obtained after optimization with the WOA algorithm exhibits a better fitting effect.

### 3.4. Case Study Results and Analysis

(1) Database establishment

Building a structural mechanical response database for asphalt pavements with varying subgrade stiffness using the approach outlined in Section 2.4.

(2) Determine matching criteria and solution objectives

Referring to China Specifications for Highway Design of Asphalt Pavement (JTG D50-2017), select maximum tensile strain at the bottom of the asphalt surface layer (*R*_1_), maximum tensile stress at the bottom of the base layer (*R*_2_), and vertical compressive strain at the top of the subgrade (*R*_3_) as matching criteria, with dynamic modulus master curve parameter combinations as the solution targets. Three sets of dynamic modulus master curve parameter combinations were randomly selected for analysis, along with their structural mechanical responses for asphalt pavement under 20 °C conditions. The specific combinations are detailed in Table 16.

(3) Parameter combination matching based on pavement structure response

**Figure 16:**
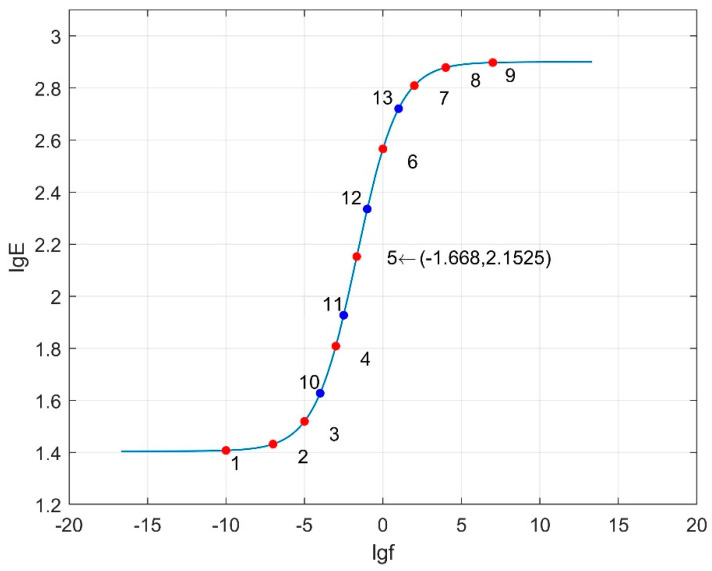
Dynamic modulus master curve (combination 1: *δ* = 1.405, *α* = 1.495, *β* = −1.246, *γ* = −0.747).

**Figure 17:**
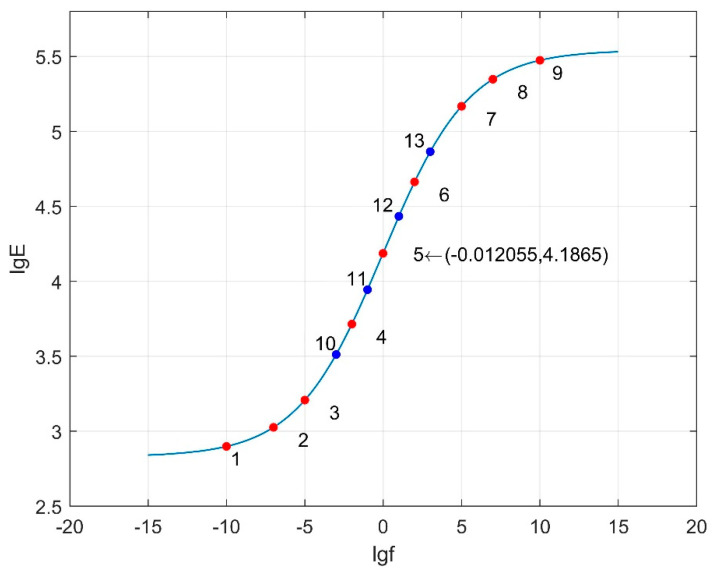
Dynamic modulus master curve (combination 2: *δ* = 2.83, *α* = 2.713, *β* = −0.0044, *γ* = −0.365).

**Figure 18:**
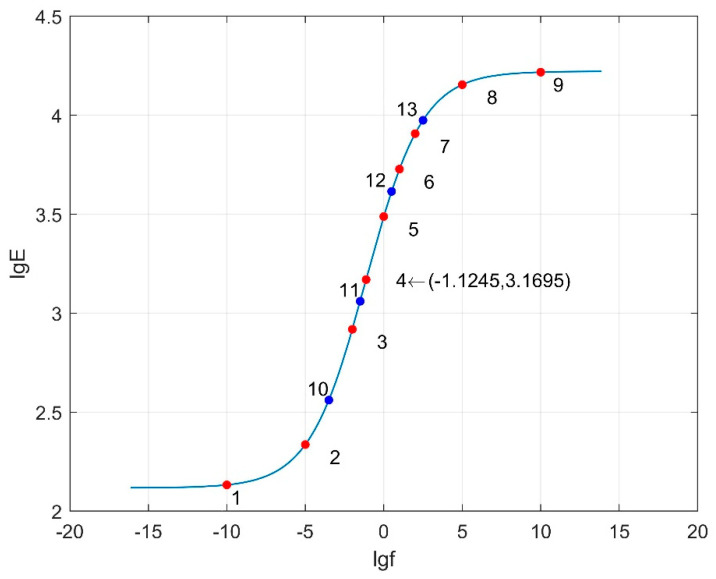
Dynamic modulus master curve (combination 3: *δ* = 2.1175, *α* = 2.104, *β* = −0.6252, *γ* = −0.556).

Figure 16, Figure 17 and Figure 18 depict the dynamic modulus master curves corresponding to parameter combinations 1 to 3. Based on the characteristics of the master curves, nine key points are selected to represent the mechanical response of asphalt pavement under different frequency conditions. Points 1 to 9, chosen from the master curves, include inflection points, upper and lower asymptotes, and other significant features, effectively capturing the main curve characteristics. Parameters *β* and *γ* respectively influence the position of the inflection point and the slope of the middle segment of the master curve. In order to more accurately capture the variations in *β* and *γ* with frequency, the vicinity of the inflection point on the master curve is densified. Therefore, four additional response values at frequencies corresponding to Points 10 to 13 are included as matching conditions. The frequency values corresponding to Points 1 to 13 are provided in Table 17, and the specific response values are calculated using finite element models.

When solving for the dynamic modulus master curve parameters based on critical structural responses, the selection of the error threshold is crucial for the accuracy and rationality of the matching. A too-large error threshold may lead to non-unique solutions (multiple sets of parameters simultaneously satisfying the matching conditions), while a too-small error threshold may result in no solution. According to the sensitivity coefficient table (Table 18), it can be observed that the sensitivity of structural responses to variations in master curve parameters varies for different asphalt pavement structures. In rigid and semi-rigid base asphalt pavement structures, the sensitivity of base bottom tensile stress to parameter variations is minimal, while in flexible base asphalt pavement structures, the sensitivity of subgrade vertical compressive strain to parameter variations is minimal. The order of magnitude for the sensitivity coefficients of the same structural response parameters is δ > α > β ≈ γ. Therefore, based on the sensitivity of asphalt pavement structural responses to fluctuations in dynamic modulus master curve parameters, the error threshold for parameter solving can be determined: Select the minimum change in the critical structural response corresponding to a 5% variation in the least sensitive parameter γ as the error threshold S_γ, 5%_, as shown in Table 18.

Setting the error threshold as *S_γ_*_, 5%_, and inputting it into the database, all queries yield a unique solution, and they are consistent with the original values, as shown in Table 19.

In conclusion, through preliminary searches, encryption screening, adjustment of error thresholds, and other steps, the matching from parameters to responses and back to parameters has been achieved, completing the dynamic modulus master curve parameter solution for three types of asphalt pavement structures. The case study demonstrates the feasibility of the asphalt mixture dynamic modulus master curve parameter design method based on pavement structure response. However, there are still issues such as an incomplete database, low accuracy of the WOA-BP prediction model, and certain errors in actual response values. These issues may lead to inaccurate results in the database. In the future, a combination of deep learning and parallel computing could be employed to further improve model accuracy and expand the database.

## 4. Conclusions

The study investigated the effects of dynamic modulus master curve parameters of asphalt mixtures and their interactions on critical structural responses using the response surface method (RSM). A WOA-BP artificial neural network was used to establish a performance prediction model. A MySQL mechanical response database was established to match master curve parameters based on critical structural responses. The main conclusions are as follows:

(1) The effects of individual asphalt mixture main curve parameters and their interactions on critical pavement responses were dependent on pavement structures. In general, parameter δ had the greatest effect on the response of each structure, followed by α, and β and γ had smaller effects. The interaction between the parameters also has a certain effect on the pavement structure response, i.e., tensile stresses at the bottom of the base.
(2) The asphalt pavement structural mechanical response prediction model was successfully established based on the BP neural network, improved by the whale algorithm.
(3) With the help of the WOA-BP neural network mechanical response prediction model, a MySQL mechanical response database was established. The methodology for determining the dynamic modulus master curve parameter based on the critical response of pavement was proposed.
(4) In the future, a larger database with more pavement types will be built in line with long-term performance observations to consistently improve the accuracy of the model. Also, more attention will be paid to building relations between the dynamic modulus master curve parameters and the asphalt mixture characteristics.

## Figures and Tables

**Table 1:** Three types of asphalt pavement structure forms.

Pavement Structure	Rigid Base	Semi-Rigid Base	Flexible Base
Asphalt Surface Layer	18 cm	18 cm	18 cm
Base Layer	30 cm Cement Concrete	15 cm + 15 cm Cement-Stabilized Crushed Stone	15 cm + 15 cm Graded Crushed Stone
Subbase Layer	20 cm	20 cm	20 cm
Subgrade	19.5 m	19.5 m	19.5 m

**Table 2:** Elastic parameters of the base course and subgrade.

Materials	Modulus (Mpa)	Poisson’s Ratio
Cement Concrete (Rigid Base)	30,000	0.15
Cement-Stabilized Crushed Stone (Semi-Rigid Base)	10,000	0.25
Graded Crushed Stone (Flexible Base)	500	0.25
Graded Crushed Stone (Subbase)	300	0.25
Subgrade	60	0.4

**Table 3:** Range of Values for Dynamic Modulus Master Curve Parameters.

Parameter	Min	Max
*δ*	1.405	2.83
*α*	1.495	2.713
*β*	−1.246	−0.0044
*γ*	−0.747	−0.365

**Table 4:** Experimental design of dynamic modulus master curve parameters.

Runs	Parameter				Runs	Parameter			
	*δ*	*α*	*β*	*γ*		*δ*	*α*	*β*	*γ*
1	2.83	2.713	−1.246	−0.365	16	2.1175	2.104	−0.6252	−0.556
2	2.1175	2.104	−1.8668	−0.556	17	2.1175	2.104	−0.6252	−0.556
3	1.405	1.495	−1.246	−0.747	18	1.405	1.495	−1.246	−0.365
4	2.83	2.713	−1.246	−0.747	19	2.83	2.713	−0.0044	−0.747
5	2.1175	2.104	−0.6252	−0.174	20	1.405	2.713	−1.246	−0.365
6	2.1175	0.886	−0.6252	−0.556	21	2.1175	2.104	−0.6252	−0.556
7	2.1175	2.104	−0.6252	−0.556	22	2.83	1.495	−1.246	−0.365
8	2.1175	2.104	−0.6252	−0.938	23	2.83	1.495	−0.0044	−0.365
9	2.1175	2.104	0.6164	−0.556	24	2.1175	3.322	−0.6252	−0.556
10	3.5425	2.104	−0.6252	−0.556	25	2.1175	2.104	−0.6252	−0.556
11	2.83	1.495	−1.246	−0.747	26	0.6925	2.104	−0.6252	−0.556
12	1.405	1.495	−0.0044	−0.365	27	1.405	2.713	−0.0044	−0.365
13	1.405	1.495	−0.0044	−0.747	28	2.1175	2.104	−0.6252	−0.556
14	2.83	2.713	−0.0044	−0.365	29	1.405	2.713	−0.0044	−0.747
15	2.83	1.495	−0.0044	−0.747	30	1.405	2.713	−1.246	−0.747

**Table 5:** Pavement structure form and material parameters.

Pavement Structure	Rigid Base			Semi-Rigid Base			Flexible Base		
	Thickness (cm)	Modulus (MPa)	Poisson’s Ratio	Thickness (cm)	Modulus (MPa)	Poisson’s Ratio	Thickness (cm)	Modulus (MPa)	Poisson’s Ratio
Asphalt surface layer	18	―	0.35	18	―	0.35	18	―	0.35
Base layer	30	30,000	0.15	30	10,000	0.25	30	500	0.25
Subbase layer	20	300	0.25	20	300	0.25	20	300	0.25
Subgrade	―	60	0.4	―	60	0.4	―	60	0.4

**Table 6:** Selection of parameters for the dynamic modulus master curve.

Parameter	Range
Parameter *δ*	1.405~2.83
Parameter *α*	1.495~2.713
Parameter *β*	−1.246 to −0.0044
Parameter *γ*	−0.747 to −0.365
Frequency *f* (Hz)	10^−4^~10^5^

**Table 7:** Neural network parameter configuration.

Parameter	Value
Maximum training iterations (times)	1000
Learning rate	0.01
Momentum factor	0.01
Minimum error	0.00001

**Table 8:** Asphalt surface layer bottom tensile strain (με).

Runs	Rigid Base	Semi-Rigid Base	Flexible Base	Runs	Rigid Base	Semi-Rigid Base	Flexible Base
1	2.62398	5.90747	12.2419	16	−5.61293	0.92218	96.4388
2	−2.38646	5.69595	65.5259	17	−5.61293	0.92218	96.4388
3	−11.6154	−18.4151	145.404	18	−11.7709	−19.2908	141.762
4	2.54743	5.18468	9.67211	19	2.03122	7.85097	25.5435
5	−7.09358	−3.19617	112.576	20	−6.19981	−1.12327	102.669
6	−11.4221	−17.5361	148.047	21	−5.61293	0.92218	96.4388
7	−5.61293	0.92218	96.4388	22	−2.13478	5.96394	63.2905
8	−4.31892	3.14004	83.4333	23	−5.74334	0.674657	97.8044
9	−10.1113	−12.3262	146.595	24	1.98584	7.88852	26.1501
10	2.59773	5.55884	10.9326	25	−5.61293	0.92218	96.4388
11	−1.48181	6.58769	57.5551	26	−12.2855	−21.6816	110.885
12	−12.2986	−21.7489	109.456	27	−10.7011	−14.6096	150.289
13	−12.1516	−21.1123	123.066	28	−5.61293	0.92218	96.4388
14	0.955492	8.03789	37.5936	29	−9.49304	−10.1366	140.455
15	−4.43294	2.96145	84.5418	30	−4.96803	2.08053	89.8375

**Table 9:** Unbound base layer bottom tensile stress (kPa).

Runs	Rigid Base	Semi-Rigid Base	Flexible Base	Runs	Rigid Base	Semi-Rigid Base	Flexible Base
1	215.167	151.045	9.75177	16	430.807	310.646	39.2467
2	369.26	268.218	31.8084	17	430.807	310.646	39.2467
3	542.864	429.719	52.1623	18	545.749	434.184	52.4877
4	200.104	135.639	7.99783	19	266.735	199.045	17.2534
5	458.451	333.166	42.4635	20	441.784	319.242	40.5281
6	539.279	424.32	51.7612	21	430.807	310.646	39.2467
7	430.807	310.646	39.2467	22	364.299	265.064	31.1786
8	406.442	292.883	36.3672	23	433.249	312.523	39.5324
9	514.636	391.057	48.9662	24	268.629	200.577	17.548
10	207.914	143.634	8.87429	25	430.807	310.646	39.2467
11	351.227	256.827	29.4886	26	555.305	449.787	53.6654
12	555.548	450.201	53.7006	27	525.683	405.198	50.2303
13	552.817	445.598	53.3287	28	430.807	310.646	39.2467
14	301.462	224.792	22.5577	29	503.102	377.435	47.635
15	408.6	294.394	36.6249	30	418.698	301.614	37.823

**Table 10:** Subgrade top vertical compressive strain (με).

Runs	Rigid Base	Semi-Rigid Base	Flexible Base	Runs	Rigid Base	Semi-Rigid Base	Flexible Base
1	−21.3032	−34.9423	−62.9223	16	−48.955	−75.254	−197.862
2	−40.1145	−61.5215	−166.258	17	−48.955	−75.254	−197.862
3	−65.3584	−116.193	−289.775	18	−65.7833	−117.741	−296.815
4	−20.0648	−32.1984	−53.4672	19	−26.5122	−43.8501	−100.832
5	−52.9777	−82.876	−212.26	20	−50.5497	−78.1476	−203.477
6	−64.8306	−114.321	−282.297	21	−48.955	−75.254	−197.862
7	−48.955	−75.254	−197.862	22	−39.4128	−60.5593	−163.577
8	−45.4313	−69.3655	−185.547	23	−49.3095	−75.8837	−199.104
9	−61.2065	−102.801	−250.02	24	−26.7334	−44.1617	−102.25
10	−20.6942	−33.6243	−58.2248	25	−48.955	−75.254	−197.862
11	−37.5756	−58.1028	−156.339	26	−67.1911	−123.152	−331.368
12	−67.2269	−123.296	−332.606	27	−62.8302	−107.696	−261.731
13	−66.8245	−121.699	−320.028	28	−48.955	−75.254	−197.862
14	−30.8173	−49.5242	−125.687	29	−59.513	−98.0916	−240.236
15	−45.7425	−69.8604	−186.64	30	−47.2007	−72.2418	−191.738

**Table 11:** ANOVA results for the regression model of *ε*_1_.

Source	*ε*_1*R*_		*ε*_1*S*_		*ε*_1*F*_	
	F-Value	*p*-Value	F-Value	*p*-Value	F-Value	*p*-Value
Model	142.89	<0.0001	29.64	<0.0001	23.69	<0.0001
A-*δ*	375.89	<0.0001	80.05	<0.0001	61.84	<0.0001
B-*α*	149.85	<0.0001	27.51	<0.0001	23.15	<0.0001
C-*β*	41.38	<0.0001	9.89	0.0042	8.87	0.0064
D-*γ*	4.46	0.0449	1.12	0.3007	0.8827	0.3565
R^2^	0.9581		0.8259		0.7912	

**Table 12:** ANOVA results for the regression model of *σ*_1_.

Source	*σ*_1*R*_		*σ*_1*S*_		*σ*_1*F*_	
	F-Value	*p*-Value	F-Value	*p*-Value	F-Value	*p*-Value
Model	128.63	<0.0001	239.18	<0.0001	220.11	<0.0001
A-*δ*	1131.24	<0.0001	2160.76	<0.0001	1882.99	<0.0001
B-*α*	456.40	<0.0001	860.70	<0.0001	788.17	<0.0001
C-*β*	129.54	<0.0001	252.24	<0.0001	206.55	<0.0001
D-*γ*	13.29	0.0024	23.65	0.0002	20.93	0.0004
AB	23.96	0.0002	3.33	0.0879	67.62	<0.0001
AC	2.81	0.1143	0.7026	0.4151	8.64	0.0101
AD	0.4438	0.5154	0.1475	0.7064	1.13	0.3049
BC	10.37	0.0057	28.64	<0.0001	19.81	0.0005
BD	0.9185	0.3531	2.26	0.1536	1.76	0.2046
CD	0.3162	0.5822	0.8046	0.3839	0.7800	0.3911
A^2^	22.93	0.0002	4.74	0.0459	61.91	<0.0001
B^2^	6.95	0.0187	0.0610	0.8083	20.62	0.0004
C^2^	1.04	0.3231	8.38	0.0111	1.20	0.2905
D^2^	0.0114	0.9164	0.1127	0.7417	0.0196	0.8904
R^2^	0.9917		0.9955		0.9952	

**Table 13:** ANOVA results for the regression model of *ε*_2_.

Source	*ε*_2*R*_		*ε*_2*S*_		*ε*_2*F*_	
	F-Value	*p*-Value	F-Value	*p*-Value	F-Value	*p*-Value
Model	153.51	<0.0001	239.36	<0.0001	6419.53	<0.0001
A-*δ*	402.36	<0.0001	631.12	<0.0001	58,274.83	<0.0001
B-*α*	161.16	<0.0001	244.77	<0.0001	24,746.77	<0.0001
C-*β*	45.76	<0.0001	74.42	<0.0001	5816.71	<0.0001
D-*γ*	4.78	0.0383	7.12	0.0132	560.69	<0.0001
AB					14.04	0.0019
AC					0.3272	0.5758
AD					0.0441	0.8366
BC					236.56	<0.0001
BD					26.08	0.0001
CD					42.24	<0.0001
A^2^					6.60	0.0214
B^2^					24.59	0.0002
C^2^					101.50	<0.0001
D^2^					1.75	0.2055
R^2^	0.9609		0.9746		0.9998	

**Table 14:** Sensitivity Coefficient Table.

	Rigid Base			Semi-Rigid Base			Flexible Base		
P	ε_1R_	ε_2R_	σ_1R_	ε_1S_	ε_2S_	σ_1S_	ε_1F_	ε_2F_	σ_1F_
δ	−3.59	−1.13	−0.89	47.19	−1.30	−0.94	−2.19	−1.00	−1.15
α	−2.77	−0.87	−0.68	37.24	−1.00	−0.72	−1.68	−0.76	−0.87
β	0.41	0.13	0.10	−4.71	0.15	0.09	0.11	0.11	0.13
γ	0.37	0.11	0.09	−4.19	0.13	0.08	0.09	0.10	0.11

Note: The data in the table represents the mean values of S under parameter variations.

**Table 15:** Neural network training results after optimization of WOA.

Structure	Rigid Base Pavement	Semi-Rigid Base Pavement	Flexible Base Pavement
Neural Network	WOA-BP	WOA-BP	WOA-BP
Training samples R	0.95792	0.96043	0.95941
Validation samples R	0.97237	0.96845	0.97639
Testing samples R	0.94742	0.96031	0.97883
Overall samples R	0.95861	0.96160	0.96487

**Table 16:** Parameter combination values.

Parameter Combination	*δ*	*α*	*β*	*γ*
1	1.405	1.495	−1.246	−0.747
2	2.83	2.713	−0.0044	−0.365
3	2.1175	2.104	−0.6252	−0.556

**Table 17:** Corresponding frequency values of feature points.

Parameter Combination	Frequency (Hz)
1	10^−10^, 10^−7^, 10^−5^, 10^−4^, 10^−3^, 10^−2.5^, 10^−1.668^, 10^−1^, 1, 10, 10^2^, 10^4^, 10^7^
2	10^−10^, 10^−7^, 10^−5^, 10^−3^, 10^−2^, 10^−1^, 1, 10, 10^2^, 10^3^, 10^5^, 10^7^, 10^10^
3	10^−10^, 10^−5^, 10^−3.5^, 10^−2^, 10^−1.5^, 10^−1.1245^, 1, 10^0.5^, 10, 10^2^, 10^2.5^, 10^5^, 10^10^

**Table 18:** Error thresholds for structural response.

Parameter Combination	Rigid Base Pavement Structure	Semi-Rigid Base Pavement Structure	Flexible Base Pavement Structure
1	0.05%	0.1%	0.05%
2	0.6%	0.25%	1%
3	0.45%	0.4%	0.5%

**Table 19:** Database output results.

Case	Original Value (Selected from Table 7)				Output (After Structural Response-Based Solving)			
Parameter Combination	*δ*	*α*	*β*	*γ*	*δ*	*α*	*β*	*γ*
1	1.405	1.495	−1.246	−0.747	1.405	1.495	−1.246	−0.747
2	2.83	2.713	−0.0044	−0.365	2.83	2.713	−0.0044	−0.365
3	2.1175	2.104	−0.6252	−0.556	2.1175	2.104	−0.6252	−0.556

## Data Availability

The original contributions presented in this study are included in the article. Further inquiries can be directed to the corresponding author.
